# Are we preparing for collaboration, advocacy and leadership? Targeted multi-site analysis of collaborative intrinsic roles implementation in medical undergraduate curricula

**DOI:** 10.1186/s12909-020-1940-0

**Published:** 2020-02-04

**Authors:** Jan Griewatz, Amir Yousef, Miriam Rothdiener, Maria Lammerding-Koeppel, on behalf of the Collaborators of the MERlin Mapping Group, Olaf Fritze, Alessandro Dall’Acqua, Mara Geißinger, Sandra Steffens, Bernhard Steinweg, Katrin Borucki, Aleksandra Germanyuk, Sarah Koenig

**Affiliations:** Competence Centre for University Teaching in Medicine, Eberhard-Karls University of Tuebingen, Baden-Wuerttemberg, Elfriede-Aulhorn-Str. 10, D-72076 Tuebingen, Germany

**Keywords:** Undergraduate medical education, Competence orientation, CBME framework, NKLM, Intrinsic roles, Curriculum development, Curriculum mapping, Reference data, Benchmarking

## Abstract

**Background:**

The Collaborator, Health Advocate and Leader/Manager roles are highly relevant for safe patient management and optimization of healthcare system in rehabilitation and prevention. They are defined in competency-based frameworks and incorporate competencies empowering physicians to master typical daily tasks in interdisciplinary, interprofessional and institutional collaboration. However, appropriate implementation of roles remains difficult in undergraduate medical education (UME) and needs to be closely monitored. The aim of this cross-institutional mapping study was to examine for the roles of Collaborator, Health Advocate and Leader/Manager: (1) To what extent do German UME programs explicitly meet the given standards after 5 years of study? (2) Which information may be obtained from multi-site mapping data for evidence-based reflection on curricula and framework?

**Methods:**

In a joint project of eight German UME programs, 80 to 100% of courses were mapped from teachers’ perspective against given national standards: (sub-)competency coverage, competency level attainment and assessment. All faculties used a common tool and consented procedures for data collection and processing. The roles’ representation was characterized by the curricular weighting of each role content expressed by the percentage of courses referring to it (citations). Data were visualized in a benchmarking approach related to a general mean of the intrinsic roles as reference line.

**Results:**

(Sub-)competencies of the Health Advocate are consistently well-integrated in curricula with a wide range of generally high curricular weightings. The Collaborator reveals average curricular representation, but also signs of ongoing curricular development in relevant parts and clear weaknesses regarding assessment and achieved outcomes. The Leader/Manager displays consistently lowest curricular weightings with several substantial deficiencies in curricular representation, constructive alignment and/or outcome level. Our data allow identifying challenges to be considered by local curriculum developers or framework reviewers (e.g. non-achievement of competency levels, potential underrepresentation, lacking constructive alignment).

**Conclusion:**

Our non-normative, process-related benchmarking approach provides a differentiated crosscut snapshot to compare programs in the field of others, thus revealing shortcomings in role implementation, especially for Leader/Manager and Collaborator. The synopsis of multi-site data may serve as an external reference for program self-assessment and goal-oriented curriculum development. It may also provide practical data for framework review.

## Background

Reforming health care by changing health professionals’ education is still a highly topical and challenging issue worldwide: Today’s “health professionals are the service providers who link people to technology, information, and knowledge” [1]. Physicians are expected to be capable of promoting patient safety and providing efficient patient care together with other healthcare professionals. Additionally, medical doctors are responsible for prevention and policymaking as collaborative leaders, respecting the expectations and needs of patients, and to ensure the community is served in an ethical and resource-economic manner in a continuously evolving health care system [2]. In response to the profound shift in the healthcare environment, competency-based medical education (CBME) has been gradually incorporated and integrated in a superordinate role framework of additional psychosocial competencies [3–5]. Role concepts, originally developed for postgraduate medical education (PME), have been defined to cover all relevant and typical facets of tasks in the daily practice of physicians. CanMEDS is only one of several frameworks, but it is the most widely adopted worldwide [5]. It specifies seven professional roles with the Medical Expert as central integrative role. All others are considered intrinsic roles [6, 7].

Although CBME concepts exist since the late 1990s [3, 8], the appropriate implementation of the superordinate roles in undergraduate medical education (UME) still represents a major challenge for medical faculties internationally [2, 4, 9–11]: This particularly matters for the Collaborator, Health Advocate and Leader/Manager. These professional roles are characterized by a high need for interaction with different people and groups within the healthcare system, thus resulting in relational complexities and intersecting sets of competencies. The roles encompass competencies empowering physicians to participate effectively and appropriately in the healthcare sector with health professional groups and institutions, with the overall aim to ensure the well-being of patients and of the population. Despite these strong interrelations each role is setting key priorities: firstly, the Collaborator is focused on preventing, negotiating and resolving interpersonal and interprofessional conflicts, thus affecting patient safety directly [12, 13]. Secondly, the Health Advocate addresses human health and well-being beyond clinical care: it emphasizes the professional contribution to collectively developing practicable concepts for change in health care system [5, 14–16]. Thirdly, the Leader/Manager deals with complex situations and scarce resources in the ongoing evolving healthcare system, envisioning doctors as a “spearhead of change” [17, 18].

In view of the special significance of the Collaborator, Health Advocate and Leader/Manager for safe patient management and optimization of healthcare system, these roles are often requested to be introduced as an integral part into UME [17]. According to the practice-based learning theory, the interrelationship of defined competencies and the way they are enacted in educational practice are highly relevant for sustainable learning oriented towards professional demands [10]. For residency, students should be fundamentally prepared for the roles, in order to take over gradually growing responsibilities in interaction with persons and institutions quicker [10, 17]. However, there is an ongoing debate about equivalent importance of competencies, required competency levels and suitable curricular interventions across UME [14]. Yet it remains unclear in large part which design, frequency and intensity of teaching, learning and assessment is most appropriate to foster the acquisition of these competencies.

Based on the above considerations, the process of roles implementation should be evaluated systematically from the beginning. Germany is at an early stage of transforming UME to CBME. Using Germany as an example, the overall aim of this study was to evaluate the status quo of developing the above three roles in UME curricula. The study was guided by the following questions: (1) To what extent do the German UME programs explicitly meet the given standards at present after 5 years of study? (2) Which information may be obtained from multi-site mapping data for evidence-based reflection on curricula and framework?

## Methods

### Setting and sample

In 2015, the National Competency-based Learning Objectives Catalogue for Undergraduate Medical Education (NKLM) was adopted in its first version as guiding framework for Germany [19]. The catalogue will be reviewed, commented and modified by end of 2020 before becoming compulsory within a new medical licensure regulation. The comprehensive framework contains 21 chapters with the professional roles based on the CanMEDs concept being introduced in the beginning (chapters 5–11). Since 2016, medical faculties from all over Germany have participated in a joint project, mapping courses of five-year undergraduate programs against the NKLM in order to visualise curricular implementation of roles and competencies, to follow-up their evolvement and to gain reference data.

The multi-site project was led by the Competence Centre of University Teaching in Medicine Baden-Wuerttemberg in Tuebingen (CCMD). A pre-study focusing on multi-site depiction of intrinsic roles in German UME, revealed differentiated role patterns regarding the varying realization of roles and programs’ agreement, thus providing a general diagnostic orientation [20]. However, further methods are required subsequently for comprehensive insight. Benchmarking approaches have shown to provide detailed curricular reference data: firstly, by linking mapping data to the given NKLM standards, secondly, by comparing own results with data from other programs to determine one’s position in the field of others [21]. Multi-site reference data at learning objectives level promises to facilitate targeted local curriculum development as well as to support the review process of the framework regarding e.g. role prioritization and topical foci.

Eight faculties (Tuebingen, Freiburg, Ulm, Hannover, Bonn, Magdeburg, Frankfurt and Wuerzburg) contributed to the current analysis of mapping data focusing on the explicit curricular representation of three professional roles: Collaborator, Health Advocate, and Leader/Manager (corresponding to NKLM chapters 8–10 [19];). In 4 of the 8 programs, 95–100% of courses were mapped. The other participating programs documented at least 80% of courses, a percentage considered to be sufficient for inclusion.

### Terminology

Local datasets of medical faculties (MF) were anonymized by consecutive numbers in random order (e.g. MF_1, MF_2). Programs and roles were characterized in an overview in Table 1. The basic organizational unit was defined as “course”, although it might be differentiated further by different lengths. A “citation” refers to any objective taught in a course and ticked off by the mapper in the mapping process. Teaching an objective on one or more occasions in a course corresponds to one citation. Competencies (C), sub-competencies (SC) and underlying objectives (O) of each intrinsic role were identified by their NKLM chapter code numbers and shortened versions of the original wordings (Table 2; for long versions refer to Additional file 1).

### Mapping software, data collection and data control

For the normalisation of the process and to ensure data quality, all faculties used the MER*lin* mapping database [22] as a common instrument and followed consented procedures, supported by hands-on instruction and individual counselling by the CCMD staff. Each faculty entered its curricular data into a separate protected data repository within the web-based MER*lin*-database application. Mapping tool, procedures as well as methods of data collection and processing have been described in detail earlier [21, 22]. A short summary is given below. Mapping of courses against given NKLM standards was conducted on sub-competency level by selecting pre-set menu options in the database: (1) the highest achieved competency level; (2) transparency in teaching (“explicit” standing for written in a study-guide, module manual or other material); (3) extent of sub-competency completeness as calculated automatically from underlying learning objectives that were ticked off in case they are taught (“objective covered”); (4) formative and/or summative or no assessment. To ensure content validity, the mapping was carried out by 47–101 faculty members per site: individuals from each discipline, often preceptors with content-related expertise of courses or senior teachers with educational background, coordinating and/or supervising courses of the department. Plausibility controls were carried out by authorized local representatives and/or staff of the dean’s offices. The global administrator of CCMD carried out regular consistency checks.

### Data processing and statistics

#### Relative weighting

Mapping data were calculated in percentages to facilitate comparison of the programs. The intrinsic roles representation was described by the curricular weighting of each objective expressed by the percentage of citations, assuming the more courses present an objective (mapping citations) the higher its curricular emphasis. To compute the curricular weighting a two-step relativization was necessary to make up for (1) site-specific and (2) framework-specific differences. Site-specific differences are displayed by the huge range of quantity of mandatory courses, respectively the amount of citations (Table 1). To achieve realistic comparability of the curricular weighting of an objective (*obj*) at a medical faculty (*MF_x*), the number of an objective’s citations (*n*_*cit*_) was put in the context of the total number of citations at that specific site for a defined reference size (*N*_*cit*_). As framework-specific differences, the varying sizes of roles (number of learning objectives in resp. chapter, *N*_*obj*_) were considered. Due to the differing numbers of objectives of roles, the resulting values were aligned with the defined reference size (*∑ roles*) to get them equally levelled and enable role comparison.

Because of the underlying concept of intrinsic roles, the reference size of relativization was theoretically set for the intrinsic roles (Note: The role of the Communicator had to be excluded, because in the NKLM the Communicator is described only on competency level and its objectives have been relocated within the framework. Thus, the consented reference size is reduced to 5 roles).

The relativized results were multiplied by a hundred, thus scaled on a percentage base: A value of hundred reliably showing average representation. The considerations led to the following formula:

$$ relative\ weighting\ \left( obj; MF\_x\right)=\frac{n_{cit\ \left( obj; MF\_x\right)}\ }{N_{cit\ \left(\varSigma\ roles; MF\_x\right)}}\bullet {N}_{obj}\ \left(\varSigma\ roles\right)\bullet 100 $$

For greater clarity in presentation of the objectives, boxplot diagrams were created with data of the eight programs, depicting the detailed weighting profile for every role (Figs. 1.1–3), although its usage is seen critically for smaller sample sizes. In the boxplot diagrams outliers were not excluded or especially emphasized. A “general mean” was implemented as a reference line to indicate high and low weightings and facilitate cross-role comparison: Assuming equal distribution of citations on objectives, multiplication of all average values by the total number of all roles’ objectives resulted in a common mean of 100 for every reference size (here: concept of intrinsic roles).

#### Degree of implementation

For analysing the actual quality of performance in teachers’ perspective, the highest competency levels achieved after 5 years of studies according to the mapping data were listed for each program in an overview in Table 1. Additionally, it was documented in how far competency parts are assessed. The site-specific specifications for the levels of objectives were compared to the NKLM requirements as given reference. The framework defines level requirements on objective level and explicitly refers to them as minimal standards. In Table 2 the number of programs that meet, exceed or fall below the standard are shown. In this study, analyses were carried out on sub-competency level to enable a greater degree of granularity without losing sight of the role perspective when being faced with comprehensive sets of objectives. For some objectives, competency level requirements were missing or heterogenous in a sub-competency: In the rare cases that level values were not given by the NKLM framework for the milestone of 5 years of study, the value of the next milestone to come was filled in. In the cases that different objective levels are given in a sub-competency, the reference value was discussed and determined in the project group. Usually the highest level reached was assumed as reference, if (1) there were other objectives on that level, and/or (2) the respective objectives were of higher relevance for the superior competency. All reference levels for learning objectives are given in the Additional file 1 for completeness. Assessments of sub-competencies were outlined roughly divided in summative and/or formative formats. If no assessment was specified for a sub-competency, this information was listed separately (Table 2).

#### Statistical analyses

Data were analysed using the Statistical Package for the Social Sciences SPSS, version 25 and Excel of the Microsoft Office Package, version 2010. The descriptive statistics including frequencies, mean, minimum, maximum, first and third quartiles were performed for faculties mapping data (citations). For cross-role comparisons the Kruskal H Test was performed. To get more precise markers for the position of an objective in relation to the general mean in the Additional file 1, a value for the tendency of representation was calculated using the Wilcoxon Signed-Ranks Test (cp. Additional file 1). The degree of agreement on objectives between programs was measured with Average Pairwise Percent Agreement.

## Results

### General role description in the UME programs

Quantitative key data about the programs’ general curricular status reveals a huge range of quantity of mandatory courses (Table 1). This is due to the heterogenous granularity of organizational structure (teaching units) within the scope of governmental regulations guaranteeing minimum standards of UME. The total number of role citations discloses that all programs explicitly represented the intrinsic roles examined; however, the Collaborator, Health Advocate and Leader/Manager clearly differed in their percentage share.

**Table 1:** Curricular status quo of the selected roles

Medical Faculty	Total number of courses (*n*)	Total number of citations (*n*)	Percentage (%)					
			Collaborator (Chap. 8)		Health Advocate (Chap. 9)		Leader/Manager (Chap. 10)	
			courses	citations	courses	citations	courses	citations
MF_1	137	495	22	46	19	33	18	21
MF_2	121	310	10	22	19	50	17	28
MF_3	178	731	26	36	23	36	25	28
MF_4	146	293	14	22	21	62	14	16
MF_5	98	473	38	34	30	38	37	28
MF_6	133	650	26	22	43	56	29	22
MF_7	102	659	36	28	38	36	34	37
MF_8	112	531	29	32	39	52	21	16

The overview includes all curricular courses at the participating faculties in which objectives of the three intrinsic roles (Collaborator Chap. 8, 24 objectives; Health Advocate Chap. 9, 18 objectives; Leader/Manager Chap. 10, 37 objectives) are addressed (citations). Data of medical faculties is presented in anonymized form (MF_1–8) and in random order

In inter-role comparison, the Health Advocate represented the highest amount of explicit citations, ranking significantly higher than the other intrinsic roles analysed (H_(2)_ = 41,514; *p* < .001). In contrast, the Leader/Manager role was mapped clearly at the lowest rate of all (mean ranks: Health Advocate 65,67; Collaborator 45,25; and Leader/Manager 24,11). These data correspond to the general impression gained from the boxplot diagrams, indicating the relative importance of the intrinsic roles and competencies (Fig. 1.1–3). The Health Advocate’s citations clearly exceed the general mean as a cross-role reference line indicating the highest degree of explicit curricular representation of the three roles. The amount citations for the Health Advocate shows a range between 33 and 62% of all citations. The mapping citations of the Collaborator’s objectives are positioned quite balanced around the general mean, ranging from 22 to 46 of percentage shares. The Leader/Manager role with its citations lies clearly below the reference line and extends from 16 to 37% of citations.

To examine the degree of programs’ matching the given NKLM role standards, the roles representation in teaching and assessment were described in detail (Table 2): (1) curricular weighting; (2) competency level achieved; and (3) presence of summative and/or formative assessment of sub-competencies. The roles are ranked based on amount of citations in descending order, starting with the Health Advocate showing the highest explicit mapping citations.

**Table 2:**
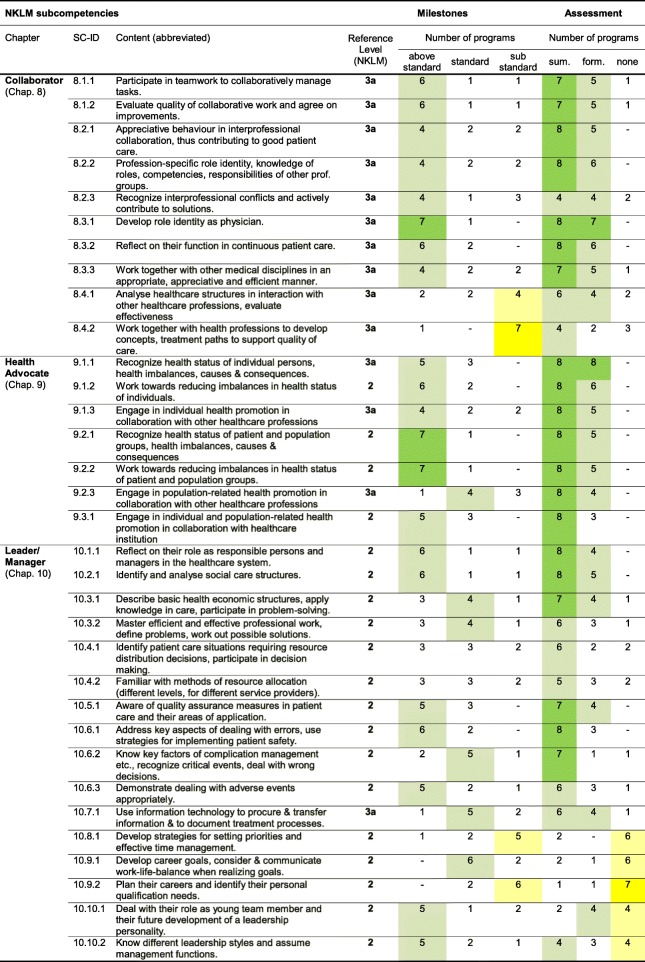
Competency level achievement and assessment of the selected roles

The Table shows the competency levels reached after 5 years of study in comparison to the given NKLM standards for sub-competencies: Level 1: knowledge/understanding/basic skills, Level 2: applied knowledge and skills in training, Level 3: competency in practice (3a: supervised, 3b: independent). Sub-competencies are specified in translations. For insight in content of superordinate competencies or sub-ordinate objectives and full-text wording see Additional file 1. Higher consent between the faculties (8–7 of eight faculties) in a column is indicated with darker colour (green = standard or above; yellow = sub-standard or none), consent above average (6–4) in lighter colour, allowing quicker diagnoses. *Sum* summative assessment; *form* formative assessment

### Health advocate

The boxplots give graphical information on the location, spread and skewness of the role’s curricular data. With very few exceptions, all learning objectives are taught intensively in all UME programs, although they show a relatively high variance in median curricular weighting and a wide spread of data regarding individual objectives (Fig. 1.2). The *SC 9.1.1 “Recognize health status of individual persons as well as health imbalances, causes and consequences”* is most strongly pronounced. Within this SC, the learning objective *O-9.1.1.3 “Identify key factors, parameters and individual resources for changing overall health situation”* is the most prominent: It shows the highest median weightings and broadly scattered values. To give an idea, the lowest value of *O-9.1.1.3* is still twice above the roles reference line.

**Fig. 1:**
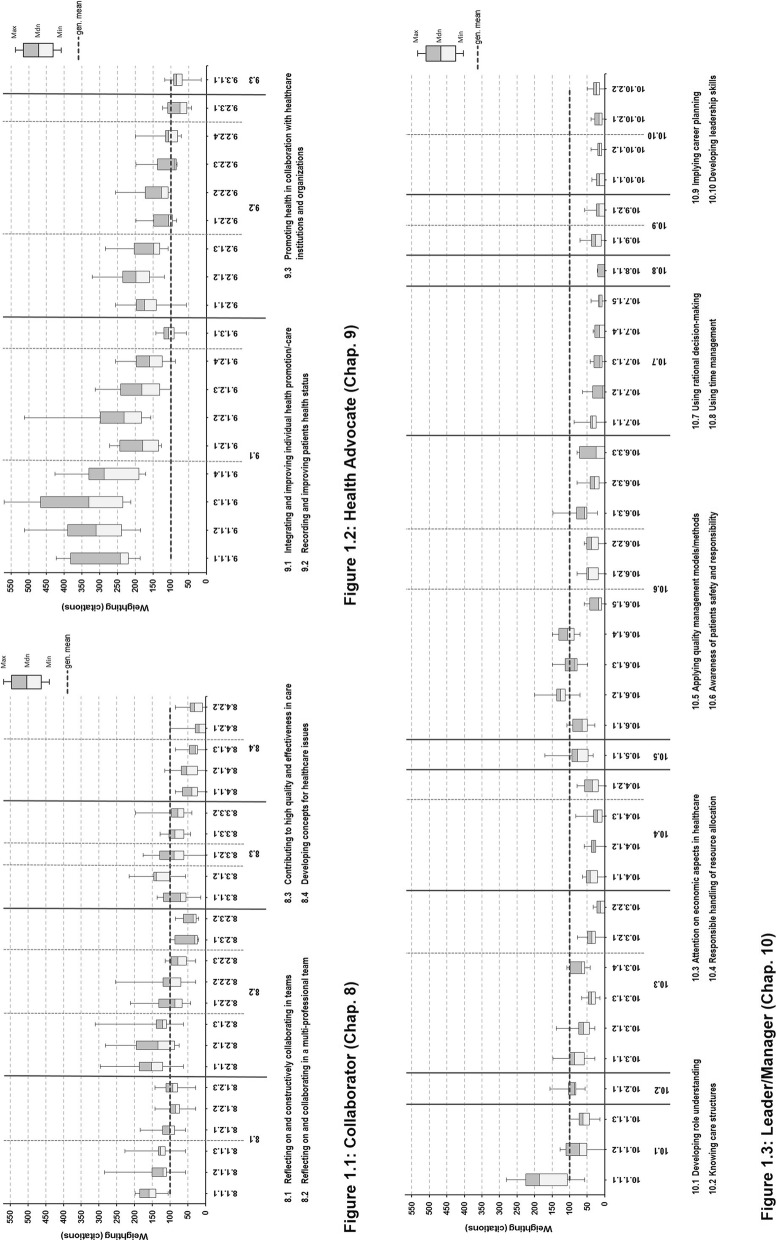
Curricular profiles of the selected intrinsic roles. In the sub-diagrams the roles of the Collaborator (Fig. 1.1), Health Advocate (Fig. 1.2) and Leader/Manager (Fig. 1.3) are displayed in boxplots. Competencies are specified in abbreviated translations. Subordinated objectives are identified by NKLM code numbers: e.g. 8.1.1.1. For insight in content and full-text wording see Additional file 1. Gen. mean = general mean of reference set

According to the mapping data (Table 2), most programs achieve an above-standard competency level for the objectives of the Health Advocate in 5 years of study. All SCs are assessed in summative formats in all UME programs and in most programs also in formative formats. Thus, this role is considered to be strongly represented and comprehensively integrated in all UME curricula.

### Collaborator

Most objectives of the Collaborator are weighted around or above the reference line, indicating this role’s respectable amount of curricular representation regarding most objectives (Fig. 1.1). Compared to the Health Advocate, the interquartile ranges appear less widely spread indicating the relatively high agreement of most programs (cp. Additional file 1). A growing number of programs appear to set a special focus on reflective collaboration in multiprofessional teams (*SC-8.2.1, SC-8.2.2*), but in a greater range of representation. However, *SC-8.2.3 “Recognize interprofessional conflicts and actively contribute to solutions”* as well as the complete competency *C-8.4. “Collaboratively develop structures, processes and concepts contributing to solve relevant healthcare issues”* display a special characteristic: they all show a noticeably low weighting, dropping even to zero. Regarding competency level (Table 2), the majority of programs achieve the minimal NKLM-standards of Level 3a (competency in practice, supervised) for this role. In most cases even a higher competency level is reached for the sub-competencies, except for the two from the low-weighted *C-8.4*. Their underlying objectives are also assessed to a lesser extent, if at all. In contrast, the higher weighted objectives of the Collaborator are otherwise assessed stronger summatively and/or formatively. Thus, it can be concluded that most objectives of the Collaborator role currently appear to be integrated with average curricular weighting and assessed correspondingly, achieving at least given minimal standard.

### Leader/manager

In comparison to the Health Advocate and the Collaborator, the Leader/Manager generally appears to be the role with the largest amount of low values in weightings, competency level and assessment. Few prominent components of this role can easily be identified in Table 2 and Fig. 1.3, because of superior or around average weighting. They reflect legal conditions and social values of the health care system and address key aspects of dealing with errors and patient safety (*O-10.1.1.1* and *O-10.6.1.2* to *O-10.6.1.4*). The competency levels intended for most of the objectives, are achieved at minimal or above standard by the greater majority of programs (Table 2; *SC-10.1.1 to SC-10.7.1*). Additionally, these objectives are assessed in summative and formative formats at all sites.

In contrast to the few well-integrated role components, most other objectives show consistently low curricular weighting at the participating faculties or in some cases even none at all (Fig. 1.3). It is particularly striking that four to seven programs achieve minimal-standard or sub-standard competency level in 5 of 16 SC, but simultaneously do not to perform any assessment (Table 2; *SC-10.8.1* to *SC-10.10.2*). This group include relevant issues of general competencies like time management, career planning, and leadership (e.g. *SC-10.8.1, SC 10.9.2, SC-10.10.2)*. In summary, the Leader/Manager role shows consented low representation in most objectives. However, a third of the role demonstrates obvious shortcomings of curricular integration (including assessment) – a finding that needs to be addressed particularly in sense of constructive alignment.

## Discussion

International experiences show that implementing the superordinate role concept in UME is not an automatic process [2, 4, 10] but needs to be closely monitored with suitable tools. There are several ways to evaluate the implementation of intrinsic roles at certain stages, mostly relying on (focus group) interviews, surveys or observation of practice of the different target groups involved. The strengths of these common approaches can be especially seen in including individual viewpoints, identification of practical needs and fundamental freedom of positioning. Mapping approaches provide another resource that can be combined with existing methods or form their basis. Mapping approaches, especially in web-based databases, enable e.g. comprehensive curricular description and visualisation, a common reference, different perspectives and scalability in focus and permanent availability.

In a previous, preliminary mapping study, multi-site curricular weightings of role’s objectives and programs’ agreement were compared in a matrix map. By applying Roger’s theory of diffusions of innovations [23], the role-specific patterns gave an orienting overview to identify the roles in various stages of curricular development [20]. In a next step, the present multi-site study provides more detailed diagnostic data focussing on the curricular status quo of the (sub-)competencies and objectives of the Collaborator, Health Advocate and Leader/Manager. These roles are all highly relevant for safe patient management and optimization of the healthcare system in rehabilitation and prevention. In overview, the Health Advocate can be highlighted as a positive example of how sub-competencies are consistently well-integrated in curricula, though in wide ranges of generally high curricular weightings. In contrast, the Collaborator role indicates average curricular representation, but reveals signs of ongoing curricular development in relevant parts, as well as obvious weaknesses regarding assessment and achieved outcome. Finally, the Leader/Manager displays the consistently lowest curricular weightings of its objectives with several substantial deficiencies in curricular representation, constructive alignment and/or outcome level [24].

The benchmarking approach with a common mapping database and consented procedures [22] applied in this study, appears an appropriate strategy to support monitoring of CBME implementation [21]. Mapping data can be applied in that way at any time during curricular developmental processes. The current data set documents a crosscut snapshot to indicate the program’s current positioning in relation to others in the field. In the context of UME, this approach allows to gain (external) reference data, to identify potential for optimization and to realize best practices. In any case, the data are considered as non-normative but descriptive in benchmarking process. However, the anonymity of the program is ensured since individual data is accessible only by the respective faculties.

### Implications of role profiles

Mapping current UME curricula against consented standards (here: the German CBME framework NKLM [19]) reveals detailed information on conformities and discrepancies between curricular reality and given standards in teaching, assessment and competency level. On the one hand, these diagnostic data can help curriculum developers to identify curricular challenges in their local program. Based on that information they can decide whether and how to deal with these problems and set priorities. Whilst, on the other hand, multi-site practice-based evidences support framework reviews by critically reflecting its content and currently valid standards for perspective adjustment. There are typical constellations of diagnostic findings down to detailed objective level, affecting both or one of the target groups. In the following, frequently occurring challenges are exemplified in increasing degree of difficulty; it is discussed how they may be interpreted and dealt with, from different perspectives.

#### Role parts exceeding given standards

The Health Advocate offers characteristic examples for this data constellation. At a first glance it appears rather unproblematic: In line with international demands [1, 14–16], this role is an essential part of the UME curricula and well-integrated in many programs, though varying in frequency and intensity. It is assessed in all programs. A closer inspection shows that many sites clearly surpass the given minimal competency level in most sub-competencies, except in *SC-9.1.3* and *SC-9.2.3* addressing interprofessional health promotion in population groups and systems. Here, few programs fall short of the desired competence level and thus give the responsible local curriculum developers cause to act. In the overall evaluation, the Health Advocate shows itself as a positive role that does not currently require immediate urgent attention except in some local curricula.

#### Non-achievement of competency level

Despite the wide range of curricular weightings, heterogeneous attainments of competency levels were mapped, in some UME programs below the minimum requirements (Table 2). Typical examples are some sub-competencies of the Collaborator role, esp. *SC-8.2.1* to *SC-8.2.3*, encompassing interpersonal skills for interdisciplinary and interprofessional collaboration. These topics are evidenced as a key aspect of successful inter-professional teams being closely related to patient safety [1, 13]. In case of sub-standard representation, sub-competencies appear to be taught rather in theory (Level 2: applied knowledge and skills in training) than in practice as specified in the NKLM (Level 3a: competency in practice, supervised). Presumably this is because of missing learning opportunities, inadequate context or impeding cultural environment [25]. After review of the NKLM framework and its re-acceptance, German curriculum developers are most likely challenged to revise and intensify UME interventions ensuring that graduates are appropriately prepared for mastering collaborative practice on day 1 of residency [10].

#### Low curricular weighting but (potentially) underrepresented

Typical examples of this characteristic feature are (sub-)competencies and objectives of the Leader/Manager. They are mapped in only few or none courses but well-consented in this lowest amount of curricular representation: e.g. *SC-10.10.1* and *SC-10.10.2* focusing leadership personality and styles as well as management functions. Internationally, collaborative leadership skills are increasingly recognized as indispensable for every physician - a fact that has already been considered in the drafting (and revision) of various national frameworks [1, 18]. Because of the well-known leadership impact on patient care and safety, these objectives are recommended to be integrated stably into UME programs for advanced medical students in their clinical years. Thus, basic competency levels achieved should be further developed in the practical year and residency [17, 26]. However, inappropriate educational context and environment as well as lacking students’ involvement could result in too rare opportunities to: (1) see role modelling (e.g. cooperative leadership), (2) reflect on the realisation and (3) practice it themselves [10]. The given representation may indicate deep-rooted curricular patterns. In view of the low curricular representation in German UME programs and the development of societal needs, both curriculum developers and framework reviewers are recommended to rethink competency levels and weightings as well as to place greater emphasis on underrepresented qualities.

#### Taught but not assessed content

Several examples illustrate missing formal constructive alignment in a considerable number of programs (e.g. in the Collaborator *SC-8.2.3* addressing interprofessional conflicts; or in the Leader/Manager: again *SC-10.10.1* and *SC-10.10.2*). There is an ongoing debate regarding the testability as well as the necessity of obligatory assessment of every competency facet, especially considering the rare opportunities to explicitly experience and practice certain role aspects in clinical context [27–29]. Faculties often perceive classical assessment methods as suboptimal for non-medical content. At the same time, unawareness and lack of familiarity with alternatives like qualitative methods lead to adherence to traditional habits. However, multifaceted assessment facilitating developmental progression of competence is crucial in CBME. More information and training are essential to “create a shared mental model of required learner’s behaviour and expected level of performance” [28] and build up “continuous, comprehensive and elaborate assessment and feedback systems” [4, 27]. Besides highlighting the role of feedback, research efforts are to be intensified for further development of additional formative assessment instruments and formats [25, 29, 30]. Thus, particularly faculty and curriculum development as well as quality management measures are required to facilitate institutional and programmatic change regarding Collaborator and Leader/Manager.

#### General accumulation of curricular weaknesses

Some framework content is characterized by clear deficiencies in the majority of programs regarding the criteria included: none or very low curricular weightings, sub-standard level attainment and missing assessment (e.g. non-specific sub-competencies from Leader/Manager field addressing time management, career planning and personal qualification needs; or Collaborator role features focusing on advanced aspects of interprofessional work). This may be caused by lack of conceptual clarity in terms of definitions, role characteristics, personal-specific and context-specific features, as systematically reviewed for the Leader/Manager [31]. Notwithstanding, some roles (esp. Collaborator, Leader/Manager) appear to be less affected by external pressure like e.g. legal regulation or politics than others (e.g. Health Advocate) during the last decades. Instead, moderate signs of increasing curricular emphasis like e.g. in the Collaborator role, seem to be based rather on internal efforts of individual programs. More than in any other case mentioned above, reviewers of the framework may seek clarification on whether, and if so to what extent, a (sub-)competency should be integrated into UME (e.g. personal-specific features of planning, system-related interprofessional collaboration). If re-affirmed, every effort for institutional change must be strengthened. If identified as inappropriate, it should be removed from UME and potentially transferred to PME.

### Limitations

Some restrictions of our approach have to be considered. Over−/underestimations of competency representation cannot be excluded despite the instruction of mappers and data quality control. Mapping data may be positively or negatively biased by certain factors: e.g. knowledge, framework terminology, perception of intrinsic roles, CBME acceptance, institutional culture and enthusiasm for teaching. Therefore, mapping data cannot be taken as hard and accurate values for curriculum depiction. It is rather an actual snapshot and crosscut of curricula in the continuously changing field of UME, which must be regularly updated to show its merit. The teachers view is an important but single-sided perspective on the explicit curriculum (taught curriculum), even though results were controlled in probability checks by Dean’s offices and senior teachers. However, the students view, addressed in another project, is relevant for a multi-perspective curriculum evaluation (learned curriculum). Regarding the graphical representation of data, the sample size is considered to be rather small for visualization in boxplots and following definite interpretation about underlying structures. Nevertheless, the display in boxplots instead of dot columns provides more clarity and enough orientation to catch an informative tendency of roles development on objective level at a glance.

## Conclusions

Faced with disillusioning international experiences, faculties are well-advised to carefully monitor the progress of the implementation of the Collaborator, Health Advocate and Leader/Manager in detail. The results must be made transparent, if the intrinsic role concept is to be beneficial. The type of data evaluation applied in this study focuses on targeted in-depth analyses of defined competencies down to objectives level, to unfold a differentiated picture of the roles’ status: Conformities, deficiencies and typical constellations within the role profiles can be identified and categorized for constructive data interpretation and informed discussions. This more detailed look is a second step in a systematic concept with continuing refinement of mapping analyses. It builds on data from a preliminary mapping approach, giving orienting insight in the stages of roles curricular diffusion and enabling the identification problematic fields by using Matrix Map analysis. In this non-normative, process-related benchmarking, multi-site reference data is generated with evidences for informed self-assessment of a curriculum and decision-making on goal-oriented, tailored development at faculties. In the ensuing discourse, local culture and contexts, such as educational resources, traditions, faculty attitudes and institutional readiness for change can be considered and related to (inter-)national requirements. Thus, in quality assurance these data from practice supports local curriculum developers and framework reviewers alike. This systematic approach can be transferred and adapted to any other framework content. The key elements for its replication in other contexts are (1) a flexible web-based database depicting the framework, (2) defined mapping procedures to create meaningful data sets and (3) commitment and integration of multiple sites referring to the same (or comparable) framework. The mapping approach promises the chance to handle the enormous amount of curricular data in a productive and resource-efficient way without putting too much pressure on participating faculties and thereby hindering the change process. In perspective, regular, transparent and graduated handling of mapping data (site-specific as well as cross-site) may foster a shared mental concept regarding given standards, expected student outcomes and educational interventions.

## Supplementary information

**Additional file 1.** NKLM Objectives of Collaborator, Health Advocate and Leader/Manager in detail. This supplement includes translated full-text wordings and weightings of the objectives of three professional roles given in the NKLM framework: Collaborator (chapter 8), Health Advocate (chapter 9) and Leader/Manager (chapter 10). Competencies are displayed in dark grey, sub-competencies in light grey. The weighting of faculties is differentiated in a) tendency (high weighting, above the general median = ➚ ; low weighting, below the general median = ➘; medium weighting, on the general median = ➔) and b) agreement between faculties (100–75 - full/good agreement =; < 75–50 - major agreement =; < 50–25 - minor agreement =; < 25 - no agreement =).

## Data Availability

The datasets analysed during the current study are not publicly available due to contract agreements. Data are available from the corresponding author in anonymized version upon reasonable request and with permission of the participating medical faculties as third party and owners of their local curricular data.

## Abbreviations

CBME: Competency-based medical education
CCMD: Competence Centre Medical Didactics (short form of: Competence Centre for University Teaching in Medicine, Baden-Wuerttemberg)
NKLM: National Competency-based Learning Objectives for Undergraduate Medical Education
PME: Postgraduate medical education
UME: Undergraduate medical education
